# Supplementary figures and images for: The interaction of p130Cas with PKN3 promotes malignant growth
Source: Mol Oncol. 2018 Dec 3;13(2):264–89. doi: 10.1002/1878-0261.12401 (PMC6360386; doi:10.1002/1878-0261.12401)

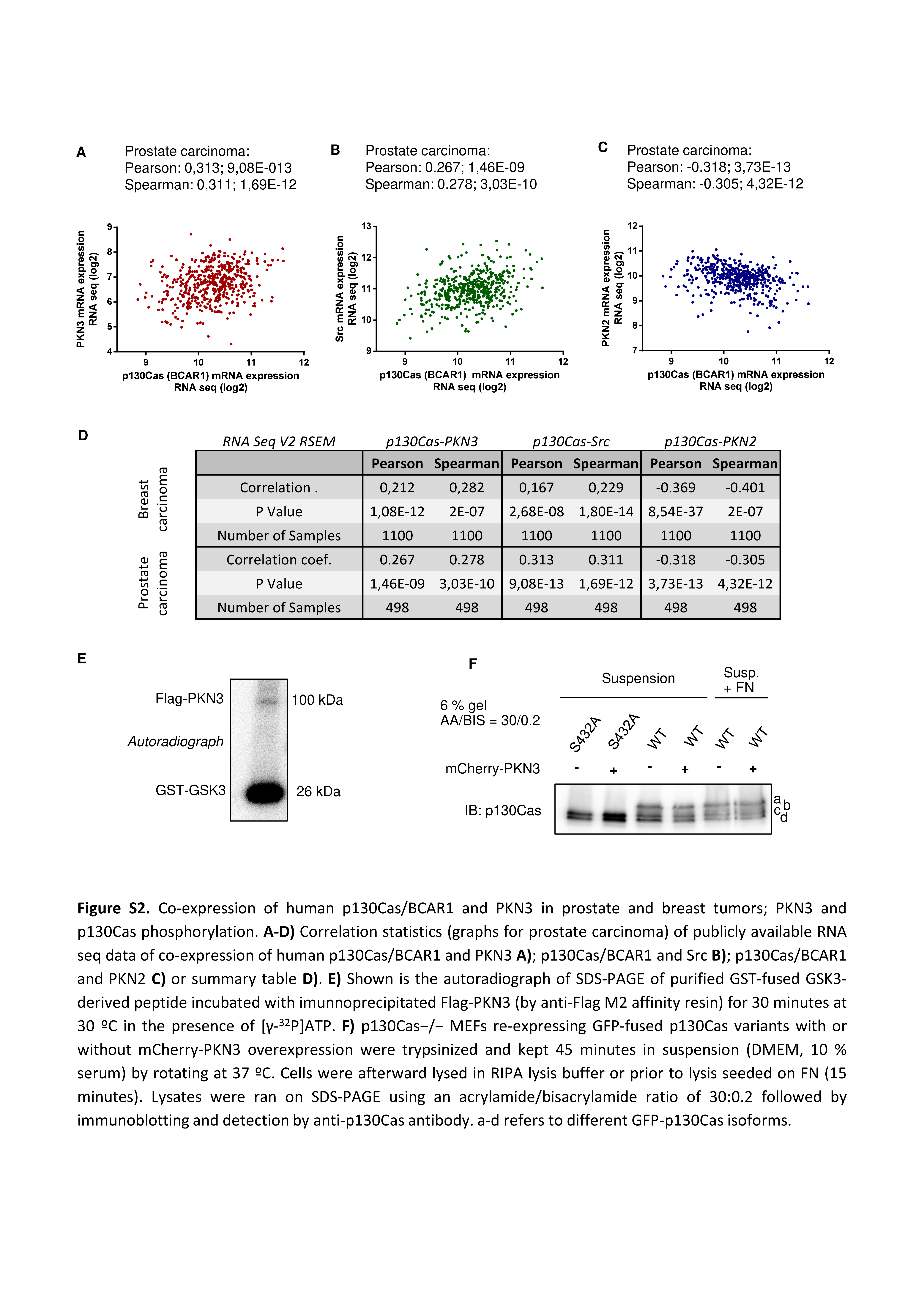

Supplement: Supplementary file 2 — Fig. S2. Co‐expression of human p130Cas/BCAR1 and PKN3 in prostate and breast tumors; PKN3 and p130Cas phosphorylation. [file MOL2-13-264-s002.tif]

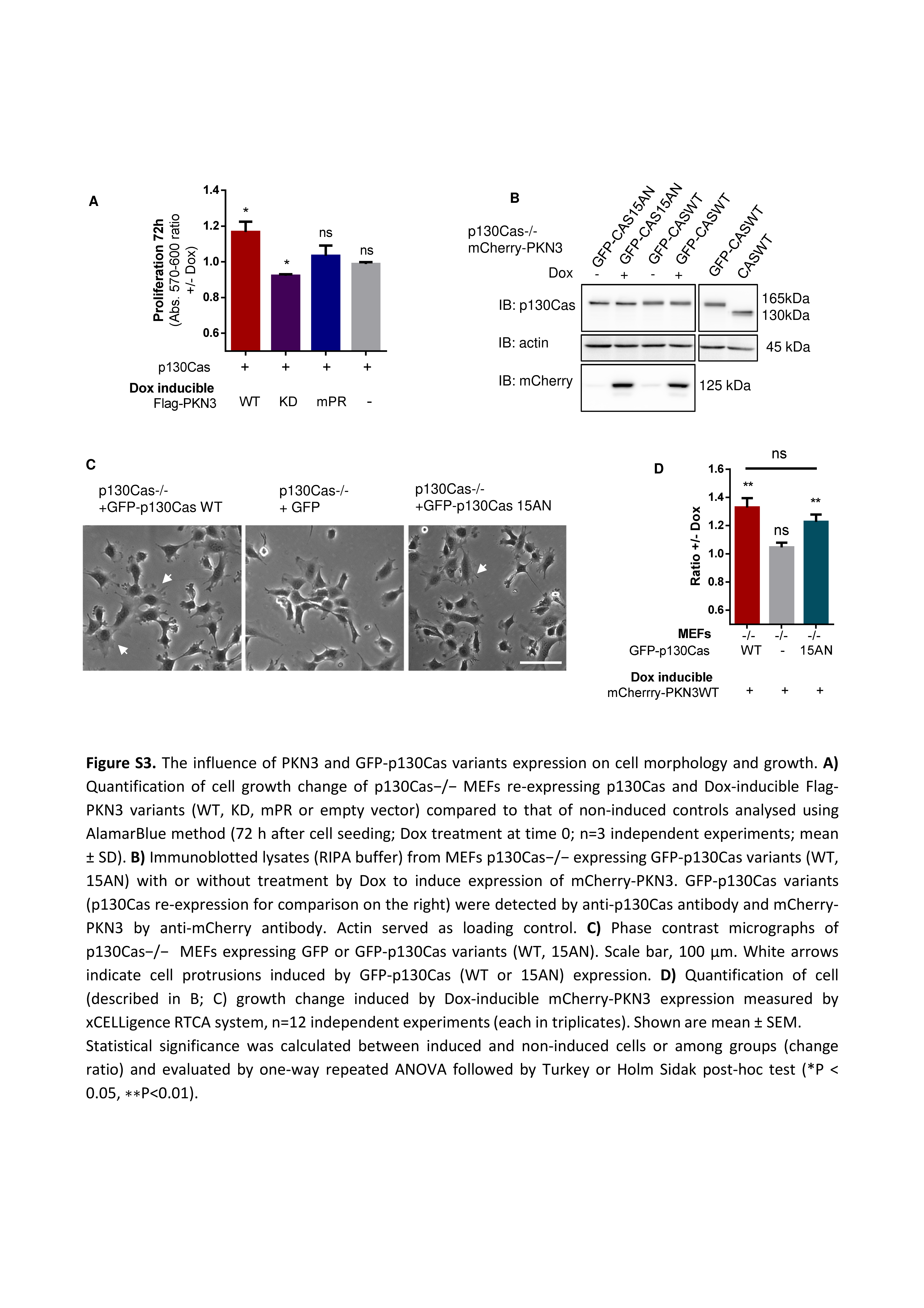

Supplement: Supplementary file 3 — Fig. S3. The influence of PKN3 and GFP‐p130Cas variants expression on cell morphology and growth. [file MOL2-13-264-s003.tif]

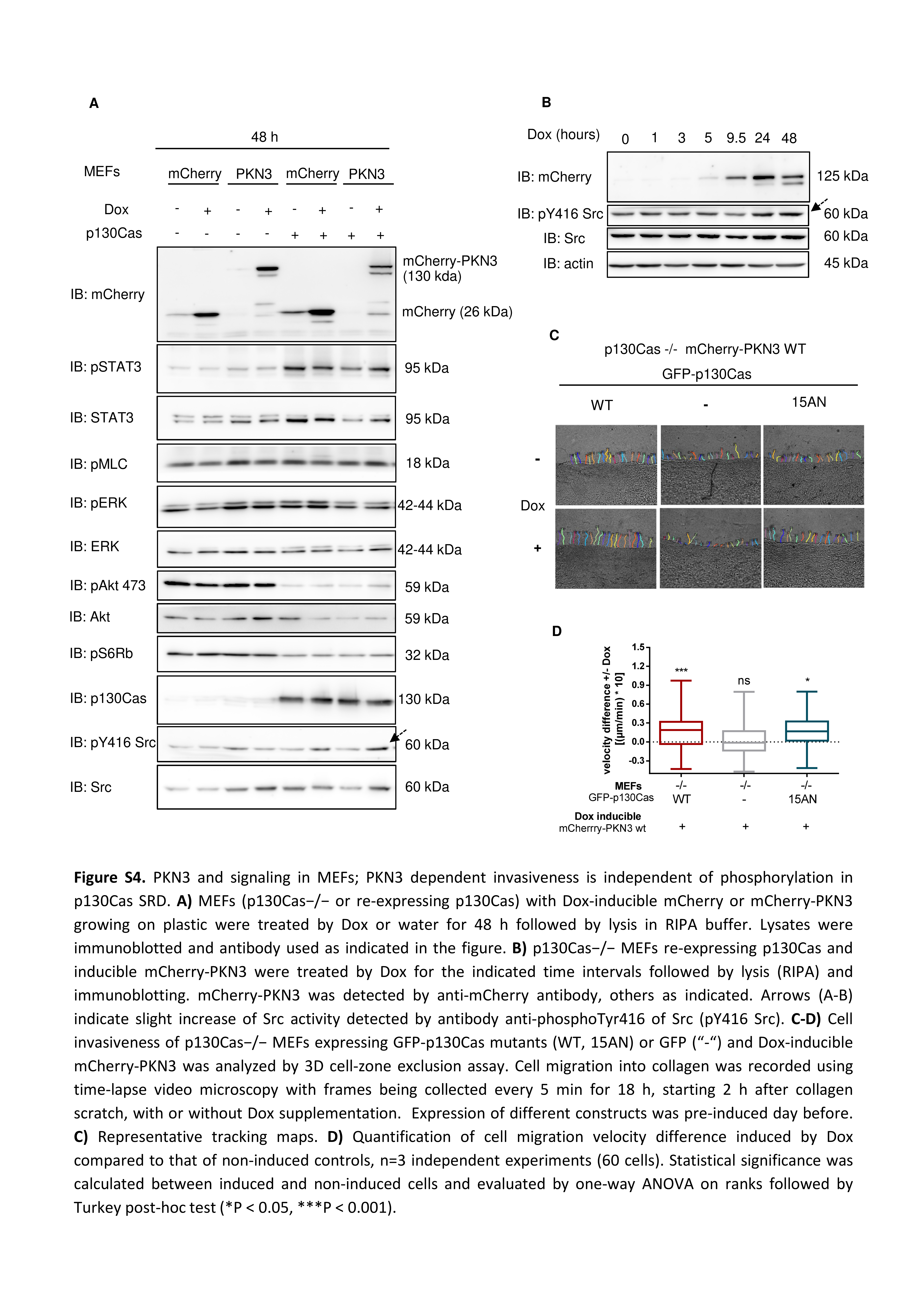

Supplement: Supplementary file 4 — Fig. S4. PKN3 and signaling in MEFs; PKN3 dependent invasiveness is independent of phosphorylation in p130Cas SRD. [file MOL2-13-264-s004.tif]

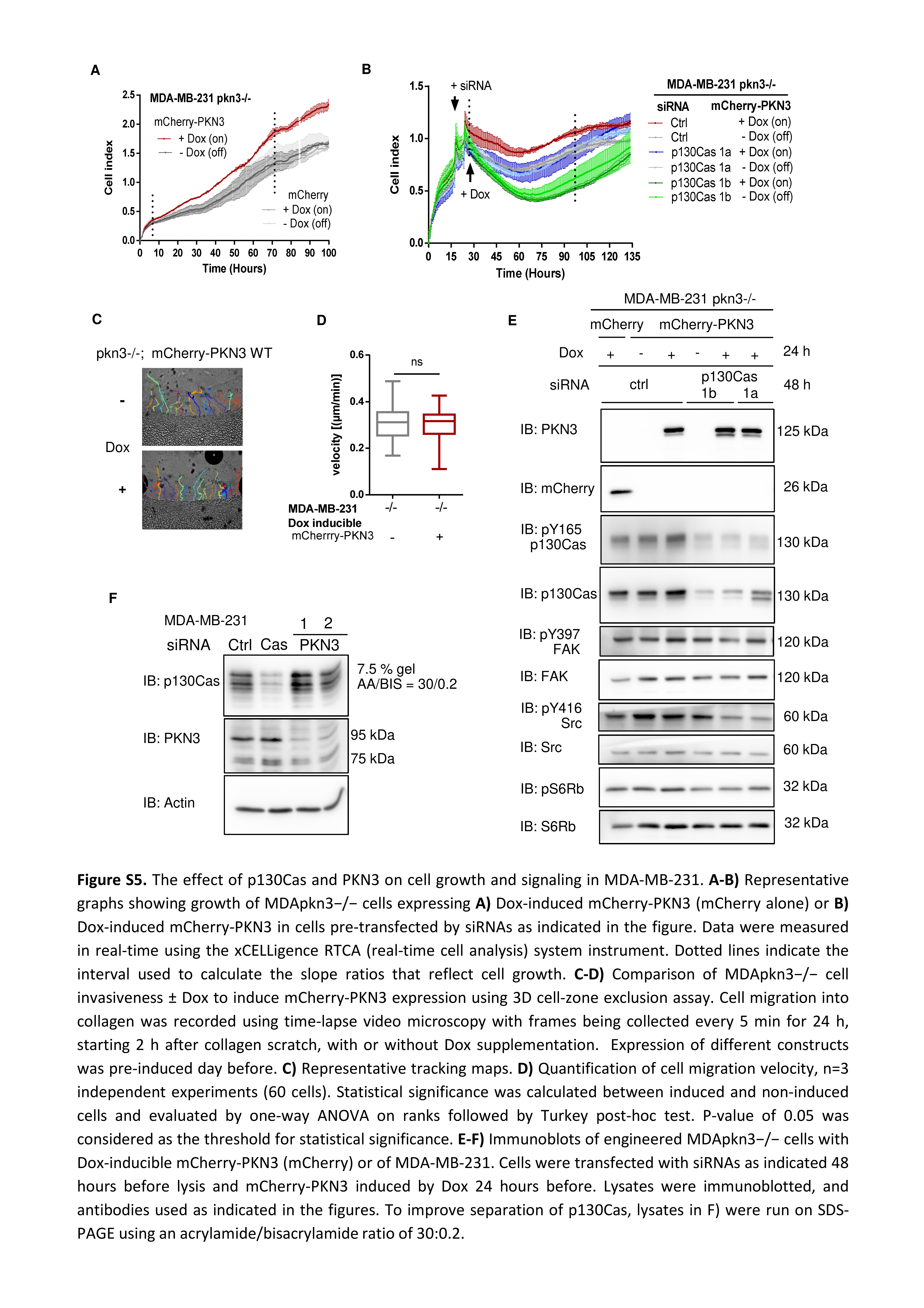

Supplement: Supplementary file 5 — Fig. S5. The effect of p130Cas and PKN3 on cell growth and signaling in MDA‐MB‐231. [file MOL2-13-264-s005.tif]

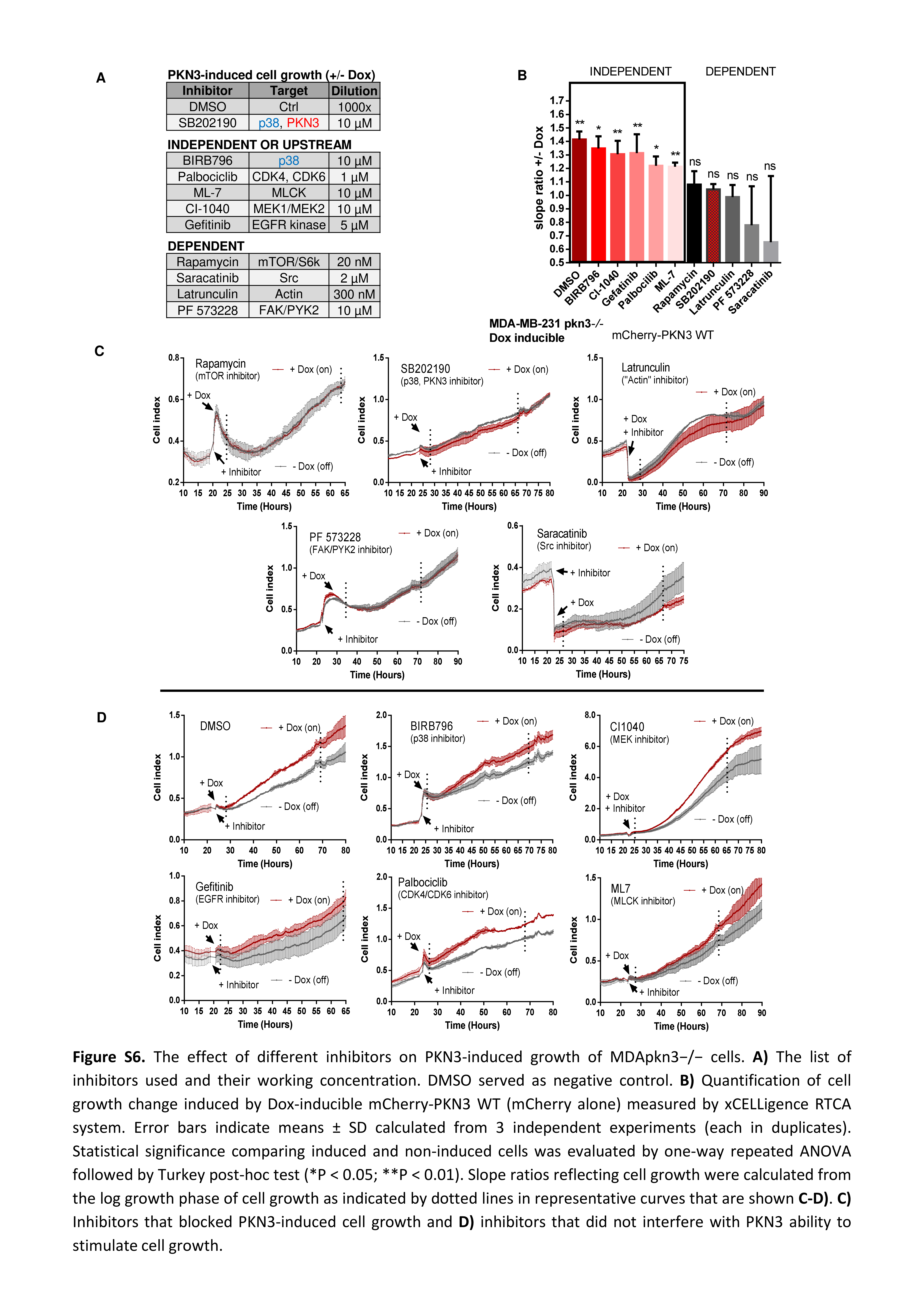

Supplement: Supplementary file 6 — Fig. S6. The effect of different inhibitors on PKN3‐induced growth of MDApkn3−/− cells. [file MOL2-13-264-s006.tif]

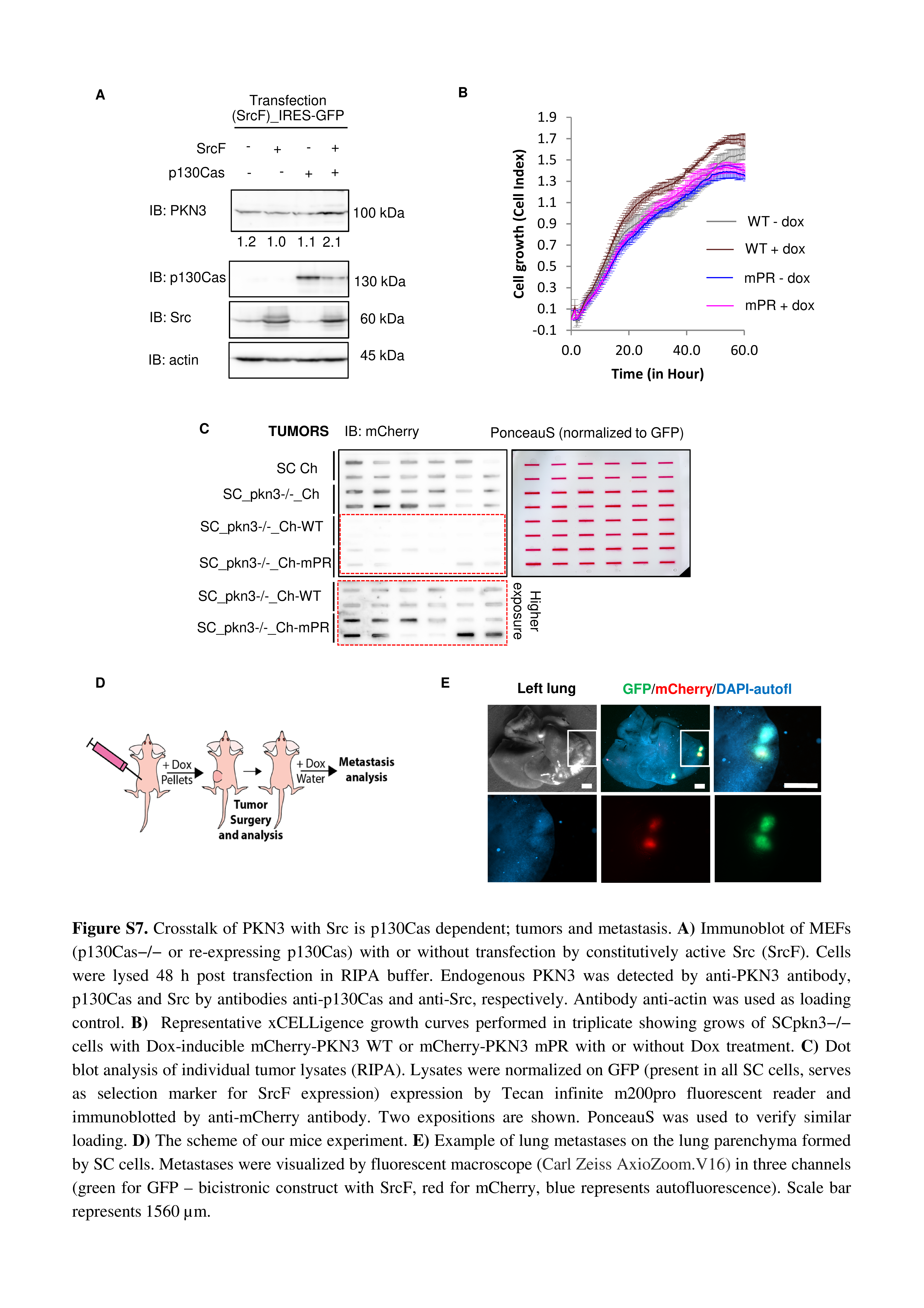

Supplement: Supplementary file 7 — Fig. S7. Crosstalk of PKN3 with Src is p130Cas dependent; tumors and metastasis. [file MOL2-13-264-s007.tif]
