# Supplementary material for: The interaction of p130Cas with PKN3 promotes malignant growth
Source: Mol Oncol. 2018 Dec 3;13(2):264–89. doi: 10.1002/1878-0261.12401 (PMC6360386; doi:10.1002/1878-0261.12401)
Supplement: Supplementary file 15 [file MOL2-13-264-s015.docx]

**Supplemental material**

Gemperle et al.,

**SUPPLEMENTAL FIGURE LEGENDS**

**Figure S1.** PKN3 colocalizes with stress fibers; PKN3 activity. **A)** Kinase reactions in vitro with precipitated mouse Flag-PKN3 WT or its KD version from transfected cells using anti-Flag M2 affinity resin. Ctrl represents immunoprecipitation from non-transfected cells. To detect PKN3 activity reactions were carried out in the presence of ATP and GST-fused peptide derived from GSK3 as described previously (Unsal-Kacmaz et al., 2011) followed by detection by anti-Phospho-GSK-3α/β antibody. Antibodies anti-Flag and GST were used to detect PKN3 kinase or GST-fused GSK3 peptide, respectively. **B)** p130Cas−/− MEFs re-expressing p130Cas were co-transfected by GFP-Flag-PKN3 variants and mCherry-LifeAct and then plated on fibronectin (FN). 24 h later cells were imaged live by Leica TCS SP2 confocal microscope system equipped with Leica 63×/1.45 oil objective**.** White arrow indicates lamellipodia and stress fibers. Corresponding or additional movies are enclosed (S1-3). **C)** p130Cas−/− MEFs or re-expressing p130Cas stimulated for mCherry-PKN3 expression were plated on FN, fixed and Stress fibers were visualized by Phalloidin (405). Cell were imaged by Nikon-Eclipse TE2000-S. Colocalized pixels (white color) above default threshold (using ImageJ) are shown. All scale bars represent 10 µm. **D)** p130Cas−/− MEFs transfected by GFP-fused p130Cas variants (WT, YE, dCCH) were lysed in RIPA buffer, blotted to nitrocellulose membrane and analyzed for endogenous PKN3 activity by antibody anti-phosphoThr849 of PKN3 (pT849 PKN3). Expression of p130Cas mutants was verified by anti-p130Cas antibody and loading by anti-PKN3 and anti-Akt antibody.

**Figure S2.** Co-expression of human p130Cas/BCAR1 and PKN3 in prostate and breast tumors; PKN3 and p130Cas phosphorylation. **A-D)** Correlation statistics (graphs for prostate carcinoma) of publicly available RNA seq data of co-expression of human p130Cas/BCAR1 and PKN3 **A)**; p130Cas/BCAR1 and Src **B)**; p130Cas/BCAR1 and PKN2 **C)** or summary table **D)**. **E)** Shown is the autoradiograph of SDS-PAGE of purified GST-fused GSK3-derived peptide incubated with imunnoprecipitated Flag-PKN3 (by anti-Flag M2 affinity resin) for 30 minutes at 30 ºC in the presence of [γ-^32^P]ATP. **F)** p130Cas−/− MEFs re-expressing GFP-fused p130Cas variants with or without mCherry-PKN3 overexpression were trypsinized and kept 45 minutes in suspension (DMEM, 10 % serum) by rotating at 37 ºC. Cells were afterward lysed in RIPA lysis buffer or prior to lysis seeded on FN (15 minutes). Lysates were ran on SDS-PAGE using an acrylamide/bisacrylamide ratio of 30:0.2 followed by immunoblotting and detection by anti-p130Cas antibody. a-d refers to different GFP-p130Cas isoforms.

**Figure S3.** The influence of PKN3 and GFP-p130Cas variants expression on cell morphology and growth. **A)** Quantification of cell growth change of p130Cas−/− MEFs re-expressing p130Cas and Dox-inducible Flag-PKN3 variants (WT, KD, mPR or empty vector) compared to that of non-induced controls analyzed using AlamarBlue method (72 h after cell seeding; Dox treatment at time 0; n=3 independent experiments; mean ± SD). **B)** Immunoblotted lysates (RIPA buffer) from MEFs p130Cas−/− expressing GFP-p130Cas variants (WT, 15AN) with or without treatment by Dox to induce expression of mCherry-PKN3. GFP-p130Cas variants (p130Cas re-expression for comparison on the right) were detected by anti-p130Cas antibody and mCherry-PKN3 by anti-mCherry antibody. Actin served as loading control. **C)** Phase contrast micrographs of p130Cas−/− MEFs expressing GFP or GFP-p130Cas variants (WT, 15AN). Scale bar, 100 µm. White arrows indicate cell protrusions induced by GFP-p130Cas (WT or 15AN) expression. **D)** Quantification of cell (described in B; C) growth change induced by Dox-inducible mCherry-PKN3 expression measured by xCELLigence RTCA system, n=12 independent experiments (each in triplicates). Shown are mean ± SEM.

Statistical significance was calculated between induced and non-induced cells or among groups (change ratio) and evaluated by one-way repeated ANOVA followed by Turkey or Holm Sidak post-hoc test (*P < 0.05, ∗∗P<0.01).

**Figure S4.** PKN3 and signaling in MEFs; PKN3 dependent invasiveness is independent of phosphorylation in p130Cas SRD. **A)** MEFs (p130Cas−/− or re-expressing p130Cas) with Dox-inducible mCherry or mCherry-PKN3 growing on plastic were treated by Dox or water for 48 h followed by lysis in RIPA buffer. Lysates were immunoblotted and antibody used as indicated in the figure. **B)** p130Cas−/− MEFs re-expressing p130Cas and inducible mCherry-PKN3 were treated by Dox for the indicated time intervals followed by lysis (RIPA) and immunoblotting. mCherry-PKN3 was detected by anti-mCherry antibody, others as indicated. Arrows (A-B) indicate slight increase of Src activity detected by antibody anti-phosphoTyr416 of Src (pY416 Src). **C-D)** Cell invasiveness of p130Cas−/− MEFs expressing GFP-p130Cas mutants (WT, 15AN) or GFP (“-“) and Dox-inducible mCherry-PKN3 was analyzed by 3D cell-zone exclusion assay. Cell migration into collagen was recorded using time-lapse video microscopy with frames being collected every 5 min for 18 h, starting 2 h after collagen scratch, with or without Dox supplementation. Expression of different constructs was pre-induced day before. **C)** Representative tracking maps. **D)** Quantification of cell migration velocity difference induced by Dox compared to that of non-induced controls, n=3 independent experiments (60 cells). Statistical significance was calculated between induced and non-induced cells and evaluated by one-way ANOVA on ranks followed by Turkey post-hoc test (*P < 0.05, ***P < 0.001).

**Figure S5.** The effect of p130Cas and PKN3 on cell growth and signaling in MDA-MB-231. **A-B)** Representative graphs showing growth of MDApkn3−/− cells expressing **A)** Dox-induced mCherry-PKN3 (mCherry alone) or **B)** Dox-induced mCherry-PKN3 in cells pre-transfected by siRNAs as indicated in the figure. Data were measured in real-time using the xCELLigence RTCA (real-time cell analysis) system instrument. Dotted lines indicate the interval used to calculate the slope ratios that reflect cell growth. **C-D)** Comparison of MDApkn3−/− cell invasiveness ± Dox to induce mCherry-PKN3 expression using 3D cell-zone exclusion assay. Cell migration into collagen was recorded using time-lapse video microscopy with frames being collected every 5 min for 24 h, starting 2 h after collagen scratch, with or without Dox supplementation. Expression of different constructs was pre-induced day before. **C)** Representative tracking maps. **D)** Quantification of cell migration velocity, n=3 independent experiments (60 cells). Statistical significance was calculated between induced and non-induced cells and evaluated by one-way ANOVA on ranks followed by Turkey post-hoc test. P-value of 0.05 was considered as the threshold for statistical significance. **E-F)** Immunoblots of engineered MDApkn3−/− cells with Dox-inducible mCherry-PKN3 (mCherry) or of MDA-MB-231. Cells were transfected with siRNAs as indicated 48 hours before lysis and mCherry-PKN3 induced by Dox 24 hours before. Lysates were immunoblotted, and antibodies used as indicated in the figures. To improve separation of p130Cas, lysates in F) were run on SDS-PAGE using an acrylamide/bisacrylamide ratio of 30:0.2.

**Figure S6.** The effect of different inhibitors on PKN3-induced growth of MDApkn3−/− cells. **A)** The list of inhibitors used and their working concentration. DMSO served as negative control. **B)** Quantification of cell growth change induced by Dox-inducible mCherry-PKN3 WT (mCherry alone) measured by xCELLigence RTCA system. Error bars indicate means ± SD calculated from 3 independent experiments (each in duplicates). Statistical significance comparing induced and non-induced cells was evaluated by one-way repeated ANOVA followed by Turkey post-hoc test (*P < 0.05; **P < 0.01). Slope ratios reflecting cell growth were calculated from the log growth phase of cell growth as indicated by dotted lines in representative curves that are shown **C-D)**. **C)** Inhibitors that blocked PKN3-induced cell growth and **D)** inhibitors that did not interfere with PKN3 ability to stimulate cell growth.

**Figure S7.** Crosstalk of PKN3 with Src is p130Cas dependent; tumors and metastasis. **A)** Immunoblot of MEFs (p130Cas−/− or re-expressing p130Cas) with or without transfection by constitutively active Src (SrcF). Cells were lysed 48 h post transfection in RIPA buffer. Endogenous PKN3 was detected by anti-PKN3 antibody, p130Cas and Src by antibodies anti-p130Cas and anti-Src, respectively. Antibody anti-actin was used as loading control. **B)** Representative xCELLigence growth curves performed in triplicate showing grows of SCpkn3−/− cells with Dox-inducible mCherry-PKN3 WT or mCherry-PKN3 mPR with or without Dox treatment. **C)** Dot blot analysis of individual tumor lysates (RIPA). Lysates were normalized on GFP (present in all SC cells, serves as selection marker for SrcF expression) expression by Tecan infinite m200pro fluorescent reader and immunoblotted by anti-mCherry antibody. Two expositions are shown. PonceauS was used to verify similar loading. **D)** The scheme of our mice experiment. **E)** Example of lung metastases on the lung parenchyma formed by SC cells. Metastases were visualized by fluorescent macroscope (Carl Zeiss AxioZoom.V16) in three channels (green for GFP – bicistronic construct with SrcF, red for mCherry, blue represents autofluorescence). Scale bar represents 1560 µm.

**Included movies are separate files relevant to the study. Movies S1-S3 show that only PKN3 WT clearly localizes to lamellipodia of p130Cas−/− MEFs reexpressing p130Cas co-transfected by GFP-PKN3 variants and mCherry-LifeActin. Movie S4-5 show (3D cell zone exclusion assay) that PKN3 increases cell migration velocity in collagen only in the presence of p130Cas.** **Tables S1 and S2 are separate Excel files that provide a list of synthetised cDNA sequences (geneArt) or oligonucleotides/siRNAs, respectively, used in this study.**

**SUPPLEMENTAL MOVIE LEGENDS**

**Supplemental Movie 1:** Colocalization of GFP-PKN3 WT and actin in lamellipodia of transfected MEF. This movie shows combined green (GFP-PKN3 WT), red (mCherry-LifeActin) and composite red/green confocal time-lapse images of transiently transfected p130Cas−/− MEFs re-expressing p130Cas plated on glass-bottom dishes (MatTek, Ashland, MA, USA) coated with 10 µg/ml FN. Cells were imaged live by Leica TCS SP2 confocal microscope system equipped with Leica 63×/1.45 oil objective**.**

Cell shows colocalization of PKN3 (on the left, GFP channel) and actin (in the middle, mCherry channel) in lamellipodia (merge on the right, green – GFP, red - mCherry). The time-lapse covers a period of about 12.5 minutes (frames being collected every 30 seconds), with approximately 4 minutes elapsed time per second of movie (VLC media player; 5.79 MB). Signal fluctuation was compensated by Stack contrast alignment in ImageJ. Scale bar represents 20 µm. Selected frame from this movie is shown in figure S1B (top).

**Supplemental Movie 2:** No colocalization of transfected GFP-PKN3 mPR with actin rich structures in lamellipodia of MEF. This movie shows combined green (GFP-PKN3 mpR), red (mCherry-LifeActin) and composite red/green confocal time-lapse images of transiently transfected p130Cas−/− MEFs re-expressing p130Cas plated on glass-bottom dishes (MatTek, Ashland, MA, USA) coated with 10 µg/ml FN. Cells were imaged live by Leica TCS SP2 confocal microscope system equipped with Leica 63×/1.45 oil objective**.** Cell shows no colocalization of PKN3 mPR (on the left, GFP channel, mCherry channel) and actin (in the middle) rich structures in lamellipodia (merge on the right, green – GFP, red - mCherry). The time-lapse covers a period of about 12.5 minutes (frames being collected every 30 seconds), with approximately 4 minutes elapsed time per second of movie (VLC media player; 7.50 MB). Signal fluctuation was compensated by Stack contrast alignment in ImageJ. Scale bar represents 20 µm.

**Supplemental Movie 3:** Filopodia-like protrusions of MEFs transfected by GFP-PKN3 KD. This movie shows combined green (GFP-PKN3 KD), red (mCherry-LifeActin) and composite red/green confocal time-lapse images of transiently transfected p130Cas−/− MEFs re-expressing p130Cas plated on glass-bottom dishes (MatTek, Ashland, MA, USA) coated with 10 µg/ml FN. Cells were imaged live by Leica TCS SP2 confocal microscope system equipped with Leica 63×/1.45 oil objective**.**

Cell shows filopodia-like protrusions. GFP channel (on the left, PKN3 KD) and mCherry channel (in the middle, LifeActin) and merge (on the right, green – GFP, red - mCherry). The time-lapse covers a period of about 20 minutes (frames being collected every 30 seconds), with approximately 4 minutes elapsed time per second of movie (VLC media player; 7.30 MB). Signal fluctuation was compensated by Stack contrast alignment in ImageJ. Scale bar represents 20 µm.

**Supplemental Movie 4:** 3D cell zone exclusion assay of p130Cas−/− MEFs re-expressing p130Cas ± Dox. These combined movies show transmitted light time-lapse images of MEFs p130Cas−/− re-expressing p130Cas with Dox-inducible mCherry-PKN3 in 3D cell-zone exclusion assay (3D collagen gel). These two movies (imaged simultaneously by two JuLI™ Br microscopes situated in the incubator) compare cell migration velocity of described cells with (on the right, 24 h Dox pre-induced) or without (on the left) treatment by Dox. The time-lapse covers a period of about 20 h (frames being collected every 5 min), with approximately 120 minutes elapsed time per second of movie (VLC media player; 9.95 MB). Selected frames (time zero) from these movies are shown in Figure 6C (on the right) with tracked 20 random migrating cells across the cell/collagen interface in the focus plane by CellTracker software.

**Supplemental Movie 5:** 3D cell zone exclusion assay of p130Cas−/− MEFs ± Dox. These combined movies show transmitted light time-lapse images of MEFs p130Cas−/− with Dox-inducible mCherry-PKN3 in 3D cell-zone exclusion assay (3D collagen gel). These two movies (imaged simultaneously by two JuLI™ Br microscopes situated in the incubator) compare cell migration velocity of described cells with (on the right, 24 h Dox pre-induced) or without (on the left) treatment by Dox. The time-lapse covers a period of about 20 h (frames being collected every 5 min), with approximately 120 minutes elapsed time per second of movie (VLC media player; 10.0 MB). Selected frames (time zero) from these movies are shown in Figure 6C (on the left) with tracked 20 random migrating cells across the cell/collagen interface in the focus plane by CellTracker software.
